# Group-Schematherapy for Adolescents: Results from a Naturalistic Multiple Case Study

**DOI:** 10.1007/s10826-016-0391-z

**Published:** 2016-03-14

**Authors:** Jeffrey Roelofs, Peter Muris, Doret van Wesemael, Nick J. Broers, Ida Shaw, Joan Farrell

**Affiliations:** Department of Clinical Psychological Science, Faculty of Psychology and Neuroscience, Maastricht University, P.O. Box 616, 6200 MD Maastricht, The Netherlands; Department of Methodology and Statistics, Faculty of Psychology and Neuroscience, Maastricht University, Maastricht, The Netherlands; Schema Therapy Institute Midwest – Indianapolis, 618 E 46th Street, Indianapolis, IN 46205 USA; Center for Borderline Personality Disorder Treatment and Research, Indiana University Purdue University, 5610 Crawfordville Rd Suite 2201, Indianapolis, IN 46202 USA; Virenze-RIAGG Maastricht, Parallelweg 45-47, 6221 BD Maastricht, The Netherlands

**Keywords:** Group schematherapy, Personality problems, Adolescents

## Abstract

Personality disorders are complex mental health problems, associated with chronic dysfunction in several life domains. Adolescents suffer from these disorders as well. The present study is a naturalistic case study, investigating whether group schematherapy (GST) can be applied to adolescents with personality disorders or personality disorder traits. Four clinically referred patients were included and completed questionnaires on quality of life, symptoms of psychopathology, schema modes, early maladaptive schemas, and schema coping styles. Patients participated in weekly GST sessions complemented by weekly or 2-weekly individual sessions. The parents of the adolescents participated in a separate parent group. From pre- to post-treatment, results demonstrated improvements for some patients in quality of life and symptoms of psychopathology. Changes in a number of modes and schemas were observed in all patients from pre- to post-therapy. In addition to assessing changes from pre- to post-treatment, the current study investigated the temporal changes in modes during therapy as well. Results demonstrated that maladaptive modes decreased, whereas healthy modes increased for all patients across the course of therapy. The present study provides preliminary support for the applicability of GST for adolescents as well as the effectiveness of GST. It is a starting point for further research on this intervention.

## Introduction

Personality disorders (PD) are complex and prevalent mental health problems. The 5th edition of the Diagnostic and Statistical Manual of Mental Disorders (DSM-5; American Psychiatric Association 2013) describes a PD as “an enduring pattern of inner experience and behaviour that deviates markedly from the expectations of the individual’s culture, is pervasive and inflexible, has an onset in adolescence or early adulthood, is stable over time and leads to distress of impairment” (APA 2013, p. 645). Prevalence rates up to 13 % have been reported for various PD’s, with the highest rates found for borderline PD (Skodol et al. 2011). PD’s are associated with chronic dysfunction in several life domains, reduced quality of life, and high societal costs (Bamelis et al. 2013).

Adolescents under the age of 18 years may suffer from PD or PD traits as well. The DSM-5 (APA 2013) allows for classification of PD’s under the age of 18 years in special circumstances. A median prevalence rate of 11 % for PD’s in adolescents has been reported (Grilo et al. 1998; Johnson et al. 2006). Research has shown that adolescents or young adults with personality disorder features suffer from increased functional impairments in later life (Skodol et al. 2007) and are at an elevated risk of suicidality and development of axis-I disorders in adulthood (Johnson et al. 1999). Therefore, there is a need for effective treatments for adolescents with personality disorder features to prevent the development of a full-blown PD.

A number of treatments for PD have been developed such as mentalisation-based treatment (e.g., Bateman and Fonagy 2004), dialectical-behaviour therapy (e.g., Linehan 1993), and schema therapy (e.g., Young et al. 2003). These treatments have different theoretical backgrounds and address personality disorder in different ways. Schema therapy (ST) has attracted wide interest and has been applied to several PD’s (Nordahl and Nysaeter 2005). ST is an integrative form of psychotherapy combining interventions from cognitive–behavioural therapy, psychodynamic therapy, gestalt therapy, interpersonal therapy, and attachment theory in one unified model. The three main concepts in ST are early maladaptive schemas, schema coping styles, and schema modes. Early maladaptive schemas consist of stable (trait-like) dysfunctional cognitions regarding oneself, one’s relationships with others, and the world. These schemas are thought to originate from the interplay between temperamental traits such as neuroticism and ongoing damaging experiences with parents, siblings, and/or peers (Young et al. 2003). The schema coping styles reflect the ways the person adapts to maladaptive schemas and to damaging childhood experiences, and represent three different styles: overcompensation (i.e., presenting oneself opposite to the maladaptive schema), avoidance (i.e., trying to avoid situations that would trigger the schema), or surrender (i.e., giving into the schema, acting as if it is true). The schema modes refer to the moment-to-moment (state-like) emotional and cognitive states and the active coping responses. Modes can explain why some individuals tend to shift rapidly in emotions and behaviors, a pattern typical for people suffering from personality disorders, like borderline personality disorder. There are four groups of schema modes. The *dysfunctional child modes* relate to the violation of basic childhood needs; *dysfunctional coping modes* refer to momentary strategies to deal with schema activation; *dysfunctionalparent modes* reflect the internalized adverse behaviors of parents (and possibly peers) towards the individual as a child. Finally, the *healthy modes* are concerned with positive and healthy cognitions as well as with behaviors (Young et al. 2003).

A number of previous studies support the effectiveness of ST, either provided in a group or an individual format, for treating PD’s in adults (Reiss et al. 2014; Bamelis et al. 2013; Farrell et al. 2009; Giesen-Bloo et al. 2006; Hoffart et al. 2002; Nadort et al. 2009; Nordahl and Nysaeter 2005; van Vreeswijk et al. 2014; Zorn et al. 2007). Systematic research on the efficacy of ST in young adults and adolescents is lacking, although there are tentative indications that this population might also benefit from such intervention (Renner et al. 2013). Since personality problems are frequently observed in young people and have been empirically linked to the underlying theoretical concepts of early maladaptive schemas, schema coping styles, and schema modes (e.g., Roelofs et al. 2015b), it seems worthwhile to examine the applicability and effectiveness of ST intervention in an adolescent population. Adolescents, in particular, might benefit from ST provided in a group format (GST), because adolescents are more focused on peers and are more likely to accept peer responses and feedback than from parents or health care professionals. Nevertheless, as most adolescents are still part of a family system, it is also considered as important to include parents or caregivers in the treatment as well.

With these issues in mind, we developed a GST program for young people with personality problems largely following the protocol described by Farrell and Shaw (2012), which included a parent component. We examined applicability and effectiveness for adolescents in a naturalistic multiple case study of four adolescents who showed clear signs of PD or PD traits (cf. Farrell and Shaw 2010). First of all, we examined the temporal change in schema modes for each of the patients during the course of therapy. It was expected that patients would learn to respond more from the healthy modes perspective and that they would display less of the dysfunctional modes. Second, a positive change was expected for quality of life, and decreases in symptoms of psychopathology, habitual schema modes, and early maladaptive schemas from pre- to post-treatment. Third and finally, the qualitative usefulness of GST was explored by interviewing each of the four patients.

## Method

### Participants

A total of four patients (three girls and one boy) were included in the current study. All patients were referred by the GP to the community mental health care centre (Virenze-Riagg Maastricht) for treatment. Each patient underwent an assessment process, which involved a standardised intake involving a semi-structured clinical interview (i.e., Kid-SCID; Hien et al. 1994; Dutch version: Roelofs et al. 2015a), and an evaluation by a psychiatrist. The outcome of the Kid-SCID interview, the psychiatrists’ evaluation information from teachers and clinical observations made during assessment were all used by the multidisciplinary team in making the final clinical diagnoses. Inclusion criteria for the GST program were suffering from personality problems (i.e., having a research classification of a personality disorder not otherwise specified), IQ >80, age >14 years, and having a structured daily life (work or school). Contraindications for participating in the GST were acute psychotic symptoms or acute suicidal behaviours. The age of the four patients ranged between 16 and 18 at time of inclusion. All patients received medication treatment at the start of GST, which was left unchanged during treatment.

#### Patient 1: Emma

Emma is a 16 year-old female who grew up in an intact family with three children. Prior to referral she had received extensive treatment at another institution where they noted depressive symptoms, sensitivity for environmental influences and persistent suicidal thoughts. That treatment consisted of psychotropic medication, and supportive and structuring treatment sessions. At time of inclusion, she was diagnosed with dysthymia, identity problems, parent–child relational problems and learning disorder not otherwise specified on axis I. She did not experience a strong bond with her father and her mother was protective towards Emma. In particular, Emma was afraid of being disapproved by others and hurting others’ feelings. On axis II she was diagnosed with a personality disorder not otherwise specified. Emma had unstable interpersonal relationships and an unstable self-image. She experienced mood changes, showed impulsive behaviors, and reported suicidal thoughts. Problems with her primary support group, problems related to the social environment and educational problems were identified on axis IV. Her global assessment of functioning score was 31–40. Both parents were involved in the treatment.

#### Patient 2: Mary

Mary is a 17 year-old female, who was referred for therapy due to relapse of depression. She grew up in an intact family with two children. Before participating in the GST program, she joined a group therapy for children with obesity and received cognitive-behavioural therapy for depression and psychotherapy for identity problems. She has been treated in our centre for 7 years with intermittent periods of no therapy. At time of inclusion, she was diagnosed with depression, identity problems, parent–child relational problems, relational problems related to a mental disorder or general medical condition and reading disorder on axis I. Mary struggled with her obesity, severe depressive symptoms, sleep problems, and an unstable self-image. She did not have peer relationships and experienced intense mood changes. She harmed herself when she felt bad and made suicidal gestures during therapy. She could not connect to her father for meeting core emotional needs and her mother tried to be available but had her own physical and mental problems. On axis II, she was diagnosed with borderline personality disorder. Problems with obesity were present on axis III. Problems with primary support group and educational problems were identified on axis IV. Her global assessment of functioning score was 41–50 at time of inclusion. Both parents were involved in the treatment.

#### Patient 3: Isabel

Isabel is an 18 year-old female who was referred for treatment for depression and self-injury. She grew up in an intact family with three children. She had previous psychological treatment but was not able to be open at that time. At time of intake, she was in her last year of secondary school. She was diagnosed with dysthymic disorder, panic disorder without agoraphobia, generalized anxiety disorder and identity problems on axis I. She felt like she could not connect to other people emotionally, had frequent suicidal thoughts and the depressive symptoms were present for longer than 1 year. There were concerns about her social and emotional wellbeing, view of the self and her minimal connection with peers. She had a low self-esteem, which was related to bullying at elementary school. At times, she would disconnect from everyone around her. On axis II the she was diagnosed with personality disorder not otherwise specified. There were no classifications on axes III and IV. Her global assessment of functioning score was 41–50 at time of inclusion. Both parents were involved in the treatment.

#### Patient 4: Josh

Josh is an 18 year-old male who was referred for GST. At time of intake, he was not in school and had little contact with his parents, because of longstanding problems within the family. He lived on his own but with assistance (i.e., someone was available to help him out). In 2013, he was admitted for 9 weeks to a mental health care institution because of alcohol dependency and abuse of cannabis and cocaine. At the beginning of 2013, he made a suicide attempt. He was diagnosed with dysthymic disorder, identity problems, parent–child relational problems and relational problems related to a mental disorder or general medical condition on axis I. Histrionic features of cluster B personality disorder were seen (i.e., dramatic behaviors and exaggerated emotional expressions), along with borderline and narcissistic features. He experienced mood changes as well. He did not have much peer contact but did have some short relationships with other (young) men. On axis II, he was diagnosed with a personality disorder not otherwise specified. There was no diagnosis on axis III. Problems with primary support group, problems related to the social environment and educational problems were identified on axis IV. His global assessment of functioning score was 41–50. His parents were musicians and their own psychiatric problems interfered with their involvement in the treatment.

### Procedure

There were two times during the year when new patients could join the group. The therapy lasted a minimum of 6 months and a maximum of 1.5 years. Four out of the six patients (and their parents), who started treatment simultaneously gave their consent to participate in the study. These four patients stayed in the group for 1 year. Eligible patients were prepared for group participation with a few individual meetings to become familiar with the schema model (schemas, modes, coping) and the schema therapy language. Before and after participating in the GST, patients completed a set of questionnaires (see *instruments*). The Therapy Session Mode Inventory was administered at the beginning of each individual treatment session and at the end of treatment.

The GST program consisted of weekly group sessions complemented by individual treatment sessions with a frequency of once per week or per 2 weeks, depending on the need of the adolescent. The individual sessions were supportive of the group sessions. That is, during individual therapy, the patient could discuss what he or she had learned or experienced in the group and there was time to talk about relevant personal issues as well. The GST largely followed the protocol by Farrell and Shaw (2012). In short, the main phases were bonding and emotional regulation, schema mode change, and finally autonomy and changing behaviour. The aim of GST was to reduce maladaptive modes and develop and strengthen functional modes. The strategies comprise specific cognitive, experiential, and behavioural techniques. Cognitive schema change work involves techniques to identify and change automatic thoughts, identify cognitive distortions, and to empirically test maladaptive rules that have been developed from schemas. Experiential interventions include work with visual imagery, mode dialogues, creative work to symbolize positive experiences, limited-reparenting and the healing experiences of a validating psychotherapist. Behavioral techniques involve pattern breaking work to ensure that changes generalize to behaviors outside of the therapy setting. During the first phase the therapeutic relationship was built. In both individual and group sessions there was a focus on building connections between the therapist and the patient, and among the patients in the group. During group sessions, there were always two therapists, one schema therapist and a creative therapist who alternately took the lead while the other followed closely interactions among the patients as well as individual responses. In this stage, limited reparenting and experiential exercises were helpful to let patients experience what it is like to focus on the experience of a feeling rather than the cognitive process of talking about feelings. Setting limits, an important aspect of limited reparenting, was utilized in this stage to make agreements on issues like self-injurious behaviour, being late, and missing sessions. During the second and third phases the focus of therapy was on the six basic needs: safety, connectedness, autonomy and individuality, realistic boundaries, expression of emotions, and spontaneity and play. Each session started with a relaxation exercise and ended with patients choosing one or several colours that fit their feelings at that moment. A number of mode focused interventions were employed in each session based upon the presenting modes and needs of the patients. If desired, patients could bring in current problem situations.

Parental involvement consisted of group meetings once every 2 weeks that were guided by two therapists who were not the GST therapists. These sessions focused on education about the schema model so that parents would be familiar with schema therapy language. The most important modes were explored in terms of ‘mode clashes’, situations where conflict occurred between parents and the adolescent. The parents were trained to be aware of their own (maladaptive) schema and schema mode activation and were given tools to use when their schema modes conflict with their child’s. Emotion coaching skills were taught, which involve being sensitive to the need of the adolescent and using emotional moments as opportunities to become more connected with the child and to teach the adolescent how to regulate emotions.

All therapists were trained in (group) ST for at least 50 h of training. The training levels of the individual therapists ranged from standard to advance in the Dutch ST Registry as well as for the International Society for Schema Therapy (ISST). All therapists were biweekly supervised by an ISST certified supervisor and there was weekly intervision.

### Measures

#### Quality of Life

The 10-item version of the Kidscreen (Kidscreen-10; Ravens-Sieberer et al. 2010) comprises one general dimension of quality of life. Each question is rated on a five-point Likert-type scale with responses reflecting the intensity of an attitude (i.e., ‘not slightly’ to ‘extremely’) or its frequency (‘never’ to ‘always’). Parents were also asked to rate the same items for their child. Higher scores indicate higher levels of health-related quality of life. The Kidscreen-10 appears to be a reliable and valid measure of quality of life in children and adolescents (Ravens-Sieberer et al. 2010).

#### Symptoms of Psychopathology

The Strengths and Difficulties Questionnaire (SDQ; Goodman 1997) is a self-report instrument designed to assess behavioral and emotional problems in children and adolescents. The SDQ was completed by the adolescent and their parent(s) (about their child). The SDQ comprises 25 items that can be allocated to five subscales: conduct problems, attention and hyperactivity problems, emotional problems, peer problems, and prosocial behavior. The four problem subscales can be combined into a total psychopathology score. Items are rated on a three-point Likert type scale with anchors ‘not true’ and ‘definitely true’, thus higher scores reflect higher levels of psychopathology symptoms. Support has been documented for the reliability and validity of the SDQ (Goodman 2001) and for the self-report version of this scale (Muris et al. 2004).

#### Schema Modes

An age-downward version of the original Schema Mode Inventory for adults (SMI; Young et al. 2007; Lobbestael et al. 2010) was used (SMI-A; Roelofs et al. 2015b). Like the adult version, the SMI-A consists of 124 items covering 14 schema modes including vulnerable child, angry child, enraged child, impulsive child, undisciplined child, happy child, compliant surrender, detached protector, detached self-soother, self-aggrandizer, bully and attack, punitive parent, demanding parent, and healthy person. Items are scored on a six-point Likert type scale ranging from ‘never or hardly ever’ to ‘always’. The overall score on the various modes can be obtained by summing the scores and dividing it by the number of items of that scale. Higher scores are indicative of a stronger presence of the modes. Psychometric properties of the SMI-A have been supported (Roelofs et al. 2015b).

#### Therapy Session Mode Inventory

For the purpose of the current study, a therapy session mode inventory was constructed. Forming a subset of all SMI-A items, this instrument comprised all schema modes by means of a single characteristic item for each separate schema mode, resulting in a total of 14 items (Roelofs et al. 2015b). For all items, respondents were required to rate on a 10-point Likert-type scale the degree to which each item (e.g. “I feel weak and hopeless”) was true for them for the last week, ranging from “applies to me completely” to “doesn’t apply to me at all”. In addition to the individual modes, three items were constructed to evaluate their relationship with their parents. These items referred to feelings of shame when talking with parents about problems, parents noticing when the adolescent is worried, and parents taking the adolescent’s feelings into account. Respondents were asked to complete this instrument during each of the individual sessions.

#### Early Maladaptive Schemas

The Young Schema Questionnaire for Adolescents (YSQ-A; Van Vlierberghe et al. 2010) is a 75-item self-report questionnaire that can be employed to comprehensively assess early maladaptive schemas in adolescents. The YSQ-A is a simplified version of the Young Schema Questionnaire for adults (YSQ; Young and Brown 2003). It assesses 15 schemas (Young et al. 2003) each represented by five items that are scored on a five-point Likert type scale with anchors ranging from ‘completely untrue for me’ to ‘describes me perfectly’. Factor analytic research (Roelofs et al. 2011; Van Vlierberghe et al. 2010) has demonstrated that the YSQ-A taps five domains of early maladaptive schemas: disconnection and rejection (including the schemas: mistrust/abuse, emotional deprivation, defectiveness/shame, social isolation/alienation, and abandonment/instability), impaired autonomy (schemas: dependency/incompetence, vulnerability to harm/illness, enmeshment/undeveloped self, and failure to achieve), impaired limits (schemas: entitlement/grandiosity and insufficient self-control/discipline), other-directedness (schemas: subjugation and self-sacrifice), and overvigilance/inhibition (schemas: emotional inhibition, unrelenting standards). Research has supported the positive psychometric qualities of the YSQ-A as satisfactory (Roelofs et al. 2011; Van Vlierberghe et al. 2010).

#### Schema Coping

The Schema Coping Inventory (SCI; Rijkeboer and Lobbestael, manuscript in preparation) assesses the three schema coping styles: overcompensation, avoidance, and surrender. The inventory consists of 12 items with each coping style represented by four items. Each item is scored on a 7-point Likert-type scale with anchors ‘completely disagree’ to ‘completely agree’. Unpublished data indicate a three-factor structure for this instrument and internal consistency.

#### Evaluation form of the GST

To evaluate the GST, an adapted version of the Schema Therapy Competency Rating Scale (STCRS; Young and Fosse 2008) was used. This version comprises 14-item tapping general therapeutic skills (e.g., limited reparenting), conceptualization and education (e.g., schema exploration and assessment), and schema change (e.g., schema strategy for change). This form was filled in after finishing the GST. Patients were required to rate on a 7-point scale the degree to which each item applied to their therapist competence, ranging from “very bad” to “excellent”.

### Data Analysis

In order to analyse the course of schema modes during therapy, we determined for each patient and mode separately whether a significant trend over time could be detected. The scores on the individual schema modes, as reflected on 10-point Likert scales, represent a time series for which we expect either negative linear trend (for the unhealthy modes) or a positive linear trend (for the healthy modes). To examine statistical evidence for the existence of these expected trends, we fitted an autoregressive time series model for each patient and for each type of mode (unhealthy vs. healthy) separately, using AR1 to model the serial correlation. Although this clearly involves multiple testing, we opted not to correct for this, as conventional methods for correcting multiple testing all have an adverse effect on statistical power. As our study is exploratory rather than confirmatory in nature, our main goal is to highlight the existence of possible trends, and therefore our primary concern here is to guard against inflation of the Type II error rate. The significant changes that were found were discussed in interviews to get a better understanding of the changes that occurred during therapy from the perspective of the adolescent. In the interviews, patients were asked how they thought that their modes have changed during therapy.

To analyse change in quality of life, psychopathology, schema modes, early maladaptive schemas, and schema coping from pre- to post-treatment, scores at both measurement points as reported by adolescents and their parents (if available) were considered in the light of norm scores or findings from previous research. To analyse quality of life, scores from the KIDSCREEN-10 were converted to *T*-scores (i.e., *M* = 50, *SD* = 10). For the SDQ, the available cut-off scores were used to determine whether observed differences between pre- and post-treatment were meaningful (see Goodman 1997). For the SMI-A, YSQ, and SCI no published normative data is available. In order to interpret change on these variables from pre- to post treatment, data from previous studies in our research group were used (i.e., Roelofs et al. 2010; Roelofs et al. 2015b; Wijk-Herberink et al. in preparation). Percentile scores were computed for the SMI-A, YSQ, and SCI, which can be obtained from the first authors. Actual scores of the four patients were compared to these percentile scores. Clinically relevant change was defined as a change of at least two decile steps (i.e., 20 % change, for example a change from percentile 50–30). In addition, for the SMI-A, we relied on normative data for various patient groups from previous research with the SMI in adults (i.e., Lobbestael et al. 2010). We first determined the change in schema modes from pre-treatment to post-treatment and compared these changes to the range of scores for the various patient groups. To analyse changes in YSQ scores from pre- to post-treatment, data obtained in non-clinical adolescents was used (Roelofs et al. 2010). For the YSQ as well, the change in scores from pre- to post-treatment was assessed and compared to the range of scores that correspond with healthy controls.

## Results

### Patient: Emma

For each separate mode, a timeseries analysis was carried out to examine the change in the mode scores over time. Table 1 (upper part) summarizes the significant findings. A significant increase was found for the Detached Self-Soother mode and the Healthy Adolescent mode, whereas a significant decrease was found for the Vulnerable Child mode, the Punitive Parent mode, and the Demanding Parent mode. In addition to these findings, data revealed that sensitivity from parents increased (i.e., talking with parents about problems made Emma feel less ashamed and parents more often noticed when she was worried about something; *t* = 2.27, *p* < .05 and *t* = 2.26, *p* < .05 respectively).

**Table 1:** Change in schema modes and pre- to post-treatment change for Emma

Schema mode change	*t*	*p*
Vulnerable child mode	−4.95	<.01
Detached self-soother mode	4.25	<.01
Punitive parent mode	−3.89	<.01
Demanding parent mode	−4.59	<.01
Healthy adolescent mode	2.28	<.05

Pre- to post-treatment change	Mean pre-treatment	Mean post-treatment
SDQ emotional problems (Emma)	7.00	3.00
SDQ hyperactivity (Emma)	8.00	5.00
SDQ total score (Emma)	25.00	15.00
SDQ hyperactivity (Mother)	6.00	5.00
SDQ total score (Mother)	14.00	10.00
SDQ hyperactivity (Father)	7.00	5.00
SDQ total score (Father)	16.00	12.00
Kidscreen (Emma)	30.00	37.00
SMI-A vulnerable child mode	6.00	1.00
SMI-A detached protector mode	5.00	1.00
SMI-A compliant surrender mode	3.43	2.00
SMI-A punitive parent mode	4.70	1.00
SMI-A demanding parent mode	3.00	1.00
SMI-A healthy adolescent mode	2.60	3.40
YSQ disconnection and rejection	5.48	2.00
YSQ impaired autonomy	3.25	2.00
YSQ need for reciprocity	5.20	2.30
YSQ need for free expression	5.20	2.00
SCI—surrender	19.00	7.00
SCI—avoidance	19.00	8.00

*SDQ* Strengths and Difficulties Questionnaire; *SMI-A* schema mode inventory for adolescents; *YSQ* Young Schema Questionnaire; *SCI* schema coping inventory

Information obtained through the interview with Emma, revealed that she confirmed most of the significant associations described above. What might have contributed to the change in the Vulnerable Child mode was that she learned to connect more with her inner feelings and that it is important not to block thoughts, feelings, and emotions. According to Emma, this might also have contributed to the positive change in the Healthy Adolescent mode and the decrease in the punitive parent and the demanding parent modes. The increase in the detached self-soother was explained by Emma in terms of the employment of more activities which brought her distraction from her inner feelings. With regard to the sensitivity from parents, Emma had experienced at start of the therapy that it was difficult for her to share emotions and experiences with her parents. However, during therapy she gradually realised that the contact and connection with her parents could be positive.

With respect to change from pre- to post-treatment, the lower part of Table 1 presents the clinically relevant findings. For symptoms of psychopathology as indexed by the SDQ, assessments of both Emma and her parents revealed reduction in symptoms of psychopathology. For quality of life, Kidscreen scores of Emma increased from more than two standard deviations below the mean score (*T* = 26.6) to less than one standard deviation below mean score (*T* = 42.1). Neither parent reported clinically relevant change so improvement was mainly reported by Emma.

Clinically relevant change in schema mode scores (i.e., change of at least two decile steps when compared to a normative sample) was found on a number of modes (see Table 1). In addition, at post-treatment, most scores were in the normative range for non-patient controls (see Lobbestael et al. 2010). Except for the schema domain of boundaries, all domains of the YSQ (early maladaptive schemas) showed a relevant change (see Table 1). At post-treatment, these scores were lower than mean scores of the non-patient group (see Lobbestael et al. 2010). Thus, with regard to change in schemas from pre- to post-treatment, results demonstrated a clinically relevant improvement on four out of five schema domains. Finally, for the SCI (schema coping), a relevant reduction in schema surrender and schema avoidance was found, indicating relevant changes in schema coping for Emma.

### Patient: Mary

Table 2 (upper part) presents the change in schema modes over time. A significant increase was found in the vulnerable child mode, the impulsive child mode, and the undisciplined child mode, whereas for the punitive parent mode both a quadratic association as well as a significant linear increase was observed. For the healthy adolescent mode a significant decrease as well as a quadratic association was observed. In addition, sensitivity from parents pertaining to feelings of shame when talking about problems increased (*t* = 6.73, *p* < .01). At the same time, a significant decrease was seen in Sensitivity from parents in terms of taking feelings into account (*t* = −2.82, *p* < .01).

**Table 2:** Change in schema modes and pre- to post-treatment change for Mary

Schema mode change	*t*	*P*
Vulnerable child mode	2.13	<.05
Impulsive child mode	3.47	<.05
Undisciplined child mode	2.88	<.01
Punitive parent mode (linear)	3.32	<.01
Punitive parent mode (quadratic)	−3.35	<.01
Healthy adolescent mode (linear)	−3.75	<.01
Healthy adolescent mode (quadratic)	3.35	<.01

Pre- to post-treatment change	Mean pre-treatment	Mean post-treatment
SDQ hyperactivity (Mary)	6.00	8.00
SMI-A impulsive child mode	2.44	2.89
SMI-A detached self-soother mode	4.50	2.75
SMI-A self aggrandizer mode	2.70	3.50
SCI—overcompensation	16.00	24.00

*SDQ* Strengths and Difficulties Questionnaire; *SMI-A* schema mode inventory for adolescents; *YSQ* Young Schema Questionnaire; *SCI* schema coping inventory

The interview with Mary provided confirmation of all of the significant associations described above. The significant quadratic effect that was observed in the Punitive parent and Healthy adolescent modes was caused by a clash in the group during therapy when new patients had entered the group. The increase in the Punitive Parent mode was due to a strong manifestation of the Punitive Parent mode halfway therapy. She explained this as a kind of inner voice telling her that she was less than others that was triggered when new patients entered the group. A decrease in the Healthy Adolescent mode was also experienced at that time. A possible explanation for the unexpected increase in the Impulsive and Undisciplined Child mode might be that the patient learned during therapy to speak up for herself even though this could sometimes hurt other people. With respect to opposite effects of therapy on two variables that index sensitivity from parents, she started sharing more with her parents about her feelings, but at the same time she realized that her parents had difficulty dealing with her feelings.

With respect to the changes from pre- to post-treatment (see Table 2, lower part), findings for symptoms of psychopathology (SDQ) demonstrated clinically relevant change but the domains of clinically relevant change were different for Mary and her mother. For quality of life, the criteria for a clinically relevant change were not met and scores from both parents were absent at post-treatment. Relevant change in scores on the SMI-A from pre- to post-treatment are shown in Table 2. SMI scores at pre-treatment revealed scores higher than mean scores of axis II patients for the Vulnerable Child mode, the Angry Child, the Enraged Child, the Undisciplined Child, the Compliant Surrender, the Detached Protector, the Detached Self-Soother, the Self-Aggrandiser, the Punitive Parent and the Demanding Parent. At post-therapy scores remained higher than mean scores of axis II patients, except scores for the Self-Soother mode, which was reduced to scores below mean scores of axis I patients. At pre- and post-treatment, scores for the Happy Child mode and the Healthy Adolescent mode were lower than mean scores of axis II patients. Scores for the Bully and Attack mode at post-treatment remained lower than mean scores of control patients. Scores for the Impulsive Child at pre-treatment were comparable to mean scores of axis I patients, but increased to post-treatment scores lower than mean scores of axis II patients (see Lobbestael et al. 2010). Taken together, most modes at post-therapy remained comparable with mean scores of axis II patients. Scores on the YSQ did not support significant change from pre- to post-treatment. Finally, for the SCI (schema coping), an increase in schema overcompensation was found.

### Patient: Isabel

With respect to the change in mode scores over time, a significant decrease was seen for the Vulnerable Child and the Detached Protector, and a borderline significant increase was found in the Happy Child mode (see Table 3). A significant quadratic association was obtained for Sensitivity from parents related to parents noticing that Isabel is worried about something (*t* = 2.49, *p* < .05).

**Table 3:** Change in schema modes and pre- to post-treatment change for Isabel

Schema mode change	*t*	*p*
Vulnerable child mode	−2.95	<.01
Detached protector mode	−3.47	<.01
Happy child mode	2.11	<.05

Pre- to post-treatment change	Mean pre-treatment	Mean post-treatment
SDQ hyperactivity (Isabel)	6.00	7.00
SDQ emotional problems (Mother)	6.00	4.00
SDQ peer problems (Mother)	4.00	2.00
SDQ hyperactivity (Father)	7.00	5.00
Kidscreen (Isabel)	28.00	36.00
SMI-A angry child mode	3.00	2.40
YSQ boundaries	4.30	2.00
SCI—avoidance	20.00	13.00

*SDQ* Strengths and Difficulties Questionnaire; *SMI-A* schema mode inventory for adolescents; *YSQ* Young Schema Questionnaire; *SCI* schema coping inventory

Information obtained during the post hoc interview with Isabel confirmed most of the significant associations described above. Although the patient did not know what might have contributed to the positive change in the Vulnerable Child mode, the Detached Protector and the Happy Child mode, the patient indicated that therapy made her stronger. With regard to the change in Sensitivity from parents, the patient had learned to share her feelings more with her parents. When new patients entered the group, she experienced difficulty in opening up to the new members and to discuss this difficulty with her parents. This might have contributed to the positive change in sensitivity from parents and explains the quadratic trend of this variable (Table 4).

**Table 4:** Change in schema modes and pre- to post-treatment change for Josh

Pre- to post-treatment change	Mean pre-treatment	Mean post-treatment
SDQ total score (Josh)	21.00	17.00
SMI-A vulnerable child mode	1.60	2.70
SMI-A compliant surrender	3.00	2.29
SMI-A punitive parent mode	1.50	2.10
SMI-A healthy adolescent mode	4.30	3.80
SCI—avoidance	14.00	19.00

*SDQ* Strengths and Difficulties Questionnaire; *SMI-A* schema mode inventory for adolescents; *SCI* schema coping inventory

With regard to change from pre- to post-treatment, symptoms of psychopathology (SDQ) decreased with more symptom reduction reported by Isabel than her parents. For quality of life a substantial improvement was reported by Isabel (i.e., an increase from three standard deviations below the mean score (*T* = 22.2) to approximately one standard deviation below mean score (*T* = 39.9). Unfortunately, quality of life scores were not available for parents. A change in scores on the SMI was found for the Angry Child mode (see Table 3). Scores on modes at pre-treatment were higher than mean scores of axis II patients for the Vulnerable Child mode, the Angry Child, the Undisciplined Child, the Compliant Surrender, the Detached Protector, the Detached Self-Soother, the Punitive Parent and the Demanding Parent. At post-treatment, these scores remained at this level except for the Angry Child mode which reduced to below the mean score of axis I patients. Scores for the Impulsive Child mode increased from a score lower than the mean score of axis II patients, to a score higher than the mean score of axis II patients. At pre- and post-treatment, scores for the Happy Child mode and Healthy Adolescent were lower than mean scores of axis II patients. Scores for the Bully and Attack mode and Self-Aggrandising mode remained at post-treatment lower than mean scores of control patients (see Lobbestael et al. 2010). Thus, these pre- to post-treatment comparisons revealed little positive change in manifestation of dysfunctional modes. In addition, at post-treatment, most modes remained comparable to the mean scores of axis II patients. Scores on the YSQ demonstrated relevant change on the domain Boundaries and for the SCI (schema coping), a relevant decrease was found for schema avoidance.

### Patient: Josh

Before addressing the results of Josh, it is important to note that he experienced a relapse in drug addiction half way therapy. He stayed in the group but this relapse clearly influenced the outcome assessments. No changes in schema modes occurred during treatment. However, a significant increase was seen in Sensitivity from parents related to taking feelings more into account (*t* = 2.32, *p* < 0.05).

Information obtained during the interview with Josh, confirmed this positive association. However, in his opinion sensitivity from parents was especially shown to the outside world and he did not feel that his parents’ expression of concern and interest was sincere.

Marginal change in symptoms of psychopathology and quality of life were found from pre to post treatment. For the SMI-A, the Vulnerable Child mode increased whereas the Compliant Surrender, the Punitive Parent, and the Healthy Adolescent modes showed a decrease. SMI-A scores were higher than the mean score of axis II patients for the Angry Child mode, the Impulsive Child, the Undisciplined Child, the Detached Protector, the Detached Self-Soother, the Self-Aggrandiser, Bully and Attack and the Demanding Parent. The post-treatment scores remained higher than mean scores of axis II patients. Scores for the Vulnerable Child mode, the Punitive Parent mode and Healthy Adolescent mode, increased at post-therapy from scores comparable to the mean score of a non-patient control group to scores comparable with mean scores of axis I patients. Scores for the Enraged child increased from higher than the mean score of axis I patients to a score higher than the mean score of axis II patients. At pre-treatment, scores for Compliant Surrender appeared to be comparable with the mean score of axis I patients and reduced to a score below the mean score of non-patient controls. Scores for the Happy Child mode remained below the mean score of non-patient controls (see Lobbestael et al. 2010). Thus, from pre- to post-treatment, some change in manifestation of modes was observed with more frequent manifestation of most modes. Scores on the domains of the YSQ did not change throughout treatment. Finally, schema avoidance was found to increase from pre- to post-treatment.

#### Patients’ Personal Evaluation of the GST

For all patients, information obtained through the evaluation form demonstrated that they felt well understood by the therapists and that therapists were able to compensate for the basic needs that were not met during childhood. The therapists were able to identify underlying patterns, modes, and coping styles and to show how these were associated with problems that the patient struggled with in everyday life. The (experiential) techniques that were used during therapy prompted them to think differently about themselves, the world, and the future. They also started to feel and act in a different way. The best working element of therapy for Emma was the individual sessions. Nevertheless, group therapy helped her to gain insight into her modes and how they could clash with those from her parents. The best working element for Mary was the exercises that were used during therapy in order to connect with feelings. For Isabel, the best working element was the exercises that focused on modes. For Josh, the best working element was that he learned to connect with his inner feelings and that he learned to show empathy to others. In addition he gained insight in his protector mode and his vulnerable child mode.

## Discussion

The primary aim of the current study was to explore whether ST is a promising therapy for adolescents with personality disorders or personality disorder traits. This study presents data from four adolescents who completed a GST program. A naturalistic study design was applied and an adapted version of the protocol employed by Farrell and Shaw (2012) was used. In summary: First, all patients showed changes in modes over therapy, but the magnitude of the change differed from one patient to another; Second, most patients showed a positive change in quality of life, symptoms of psychopathology, schemas and modes from pre-to post-treatment, although of different magnitude; Finally, all patients were able to reflect on the therapy and to indicate what aspects of therapy were most helpful to them.

With respect to changes in modes over the course of ST, the findings of the current study provide some support for the hypothesis that during therapy patients learn to respond more from a Healthy Adolescent mode perspective and less from dysfunctional modes. Although, this study did not find increases in the Healthy Adolescent mode for all patients, positive change in at least one dysfunctional mode was found in all patients. The modes that changed during treatment were different for each patient. This supports the assumption that change in schema modes from treatment will be different for each patient depending upon his/her context and the problems that he or she is dealing with. There was a significant change in sensitivity from parents for all patients and this change was positive in most cases. This finding underscores the importance of having parents involved in the therapy. Parents learned to be aware of their own dysfunctional schemas and schema modes and how and when they clash with the modes of their child. They learn to act as a coach in regulating emotions for their children. There were individual differences in the amount of change in patients; most improvement was seen for patients whose parents were involved and were able to become more sensitive to the needs of their child.

With regard to observed changes in quality of life, symptoms of psychopathology, schemas and modes, findings were only partly in line with our hypothesis. The expected change in these measures was not observed for all patients. Two patients (Emma and Isabel) showed clinically relevant improvement on a number of these measures, while the other two (Mary and Josh) didn’t attain as much gains during therapy. For Josh, his minimal gains might be explained by his relapse in drug addiction half-way through therapy, which interfered with attendance at both GST and individual sessions. A possible explanation for the lower gains of Mary might be the long-standing and resistant to change nature of her problems compared to those who benefitted more from this treatment. Outpatient treatment might not have been intensive enough to significantly impact Mary’s problems. Indeed, after the study she was referred to an inpatient treatment program.

With respect to change in schema modes, schemas, and schema coping during treatment, more change was observed in schema modes as compared to schemas. A possible explanation for this might be that some items of the schema questionnaire are formulated in a way that they are unlikely to change because these items are referring to experiences in the past (Renner et al. 2013). Further, the finding that schemas are more stable than modes, is in line with previous research demonstrating the stability of schemas over time (Riso et al. 2006; Renner et al. 2013). Modes, in contrast to schemas, are conceptualized as the current state a person is in, therefore by definition a more relevant measure of change. It remains to be determined whether change in early maladaptive schemas is necessary for symptom reduction and improvement in quality of life or that change in mode and coping style are equally relevant.

It is noteworthy that an increase in what could be considered dysfunctional modes (i.e., Impulsive Child and Vulnerable Child modes), was observed in some of the patients. However, when placed in context related to these patients, these modes might not be solely dysfunctional. An increase in the impulsive child mode was observed for Mary, who was often excessively worried about how others evaluated her. In this case acting on impulse could represent a necessary developmental step toward healthy expression. Related to the Vulnerable Child mode, it is important for someone who is not aware of his or her painful or uncomfortable feelings to become aware. This increased awareness could be reflected in higher scores as the Vulnerable Child mode would be temporarily more present. In this case, an increase in the Vulnerable Child mode can also be viewed as a positive development. In adult patients with BPD higher VCM scores were found immediately after group treatment, but decreased at 6 month follow-up (Farrell, personal communication, December 2, 2015).

Finally, it was observed that changes reported by parents were not always consistent with changes reported by patients. Changes reported by parents tended to be somewhat more positive than those reported by their children. One explanation for this might be that most of the problems adolescents are struggling with are internalising in nature, and it is well-known that such problems are less noticeable to others (Achenbach et al. 1987).

The post hoc interviews suggested that most patients could give appropriate explanations for what they thought contributed to their positive changes in schemas and modes as assessed by questionnaire. All patients felt well understood by their therapists and confirmed that therapists had identified their underlying schema patterns and modes. In addition, patients felt that therapists could, in general, compensate for the basic needs that were not met during childhood. Most patients experienced that because of the techniques that were used, they started to think differently about themselves, the world and the future. Patients clearly differed in terms of which elements of the treatment were most helpful for them.

### Limitations

Admittedly, the current study suffers from a number of limitations. First, we only described four cases of adolescents who received GST and no control group or control condition was included. Therefore, it remains uncertain whether changes are due to treatment or to non-specific factors such as the therapeutic relationship, attention from group members, or time-effects. Given that the treatment lasted for 6 months to 1 year, spontaneous improvement or maturation might be of greater concern than for case studies where the treatment is of shorter duration. The use of a naturalistic treatment design enabled us to explore the effects of treatment in an ecologically valid way, but future research should evaluate the effects of GST in a more controlled manner. Second, we did not asses the long-term effects of the group intervention, thus we cannot draw any conclusions about whether the gains were maintained after termination of therapy. Third, we relied entirely on self-report measures. Future research should include structured interview assessments and ratings of treatment adherence. Finally, another limitation is the lack of formal norms or cut-off scores for the adolescent versions of the questionnaires utilized. Although we used the results available from previous research and conducted analyses to compare adolescent means to those of existing samples, it was not possible to indicate whether gains or declines in our patients were indeed clinically significant.

Despite the aforementioned limitations, the present study adds to the emerging body of evidence, showing that GST, a promising treatment for adults, might also benefit young people with personality disorders or personality disorder features. Our findings that GST in combination with individual treatment sessions can be effective in changing symptoms of psychopathology, schemas and modes, replicates previous findings (van Vreeswijk et al. 2014; Farrell et al. 2009; Renner et al. 2013). The cost-effectiveness of GST compared to the more intense and long-term individual treatment and the potential for making a specialized treatment more available, adds to its potential value for adolescents. Although further evaluation of this model with a larger sample size and under more controlled conditions is warranted, the present study should be considered as a preliminary exploration of the effects of ST for patients under the age of 18 years with personality problems. The findings suggest that ST warrants further examination under more controlled conditions and with a larger sample as a treatment approach for the adolescent population.
